# Responses of the blood acid-base balance and blood plasma metabolomics of broiler chickens after change to diets with high free amino acid levels

**DOI:** 10.1016/j.psj.2024.103956

**Published:** 2024-06-06

**Authors:** Ahmad Ibrahim, Ákos Kenéz, Jens Pfannstiel, Iris Klaiber, Markus Rodehutscord, Wolfgang Siegert

**Affiliations:** ⁎ Institute of Animal Science, University of Hohenheim, 70599 Stuttgart, Germany; † Department of Infectious Diseases and Public Health, College of Veterinary Medicine and Life Sciences, City University of Hong Kong, Kowloon, Hong Kong SAR, China; ‡ Core Facility Hohenheim, University of Hohenheim, 70599 Stuttgart, Germany; § Department of Animal Sciences, University of Göttingen, 37077 Göttingen, Germany

**Keywords:** acid-base balance, free amino acid, broiler chicken, acidosis, metabolomics

## Abstract

Free amino acids (**AA**) are needed to fulfill the AA requirements of broiler chickens in diets low in CP. This study investigated whether the acid-base balance and the blood plasma metabolome are affected immediately after a change to diets with high free AA levels. Male broiler chickens received a starter diet with 164 g CP/kg and 80 g soy protein isolate/kg until d 7 post-hatch. From this day on, birds were offered a diet almost identical to the starter diet (**0FAA**) or 2 diets with 50% (**50FAA**) or 100% (**100FAA**) of the digestible AA from soy protein isolate substituted with free AA. Blood was sampled to determine the acid-base status and for untargeted metabolomics analysis on d 0, 1, 2, 4, 7, and 14 and d 1, 7, and 14 after diet change, respectively (n = 14 birds/treatment). Compared to 0FAA, blood pH was decreased on d 4 and 7 for 100FAA and on d 4 for 50FAA (*P* ≤ 0.019). On d 4, 7, and 14, bicarbonate, base excess, and total carbon dioxide were lower for 100FAA than for 0FAA (*P* ≤ 0.006). The partial pressure of carbon dioxide was higher for 50FAA than for 0FAA on d 4 (*P* = 0.047). Compared to 0FAA, chloride was higher for 100FAA on d 1, 2, 4, 7, and 14, and for 50FAA on d 1, 2, and 4 (*P* ≤ 0.030). In the metabolomics assay, 602, 463, and 302 metabolites were affected by treatment on d 1, 7, and 14, respectively (*P* < 0.050), but they did not indicate that metabolic pathways were affected. Flavonoids were the most consistently affected category of metabolites. The results indicated a metabolic acidosis for 100FAA from d 4 to 7 and a respiratory acidosis for 50FAA on d 4 after diet change. These types of acidosis were compensated later on in the experiment. The metabolomics analysis did not indicate that high free AA inclusion affected metabolic pathways.

## INTRODUCTION

Reducing dietary CP in poultry feeding is aimed by nutritionists to decrease nitrogen (**N**) emissions and to increase the efficiency of the conversion of protein in plant-based feed to protein in animal-based food. The use of free amino acids (**AA**) is a helpful tool to fulfill the AA requirements of broiler chickens when dietary CP is reduced. In principle, dietary AA concentrations can also be adjusted by selecting and combining feed ingredients other than free AA with favorable AA profiles, but the potential of this possibility to reduce dietary CP below current standards is limited. The main reason is the unavailability of feed ingredients with suitable AA profiles. This limits the possibility of meeting the requirements of several AA simultaneously while using free AA allows for adjustments of dietary concentrations of each AA. Free AA have enabled a considerable potential to reduce dietary CP below the current standard of 20 to 23% in the first 21 d post-hatch to 16% without performance loss (Hofmann et al., 2019; Siegert and Rodehutscord, 2019). These low CP diets contained a number of 10 free AA, which summed up to 21.5 g of free AA/kg while current practical diets usually contain less than 8 g free AA/kg of diet (Dunmire et al., 2024).

The physiological value of free and digestible peptide-bound AA may not be equivalent. A previous experiment on broiler chickens investigated an incremental substitution of peptide-bound AA from soy protein isolate (**SPI**) with free AA in 16% CP diets (Ibrahim et al., 2024a). This substitution covered all 20 proteinogenic AA and considered the different prececal AA digestibility of SPI and free AA so that the calculated supply with prececally digestible AA was identical in all treatments. As a result, ADG and ADFI were impaired when the level of free AA inclusion was increased from 54 to 71 g/kg of diet. The reduction in ADG at high AA substitution probably was mainly caused by the lower ADFI, but causes for lower ADFI were difficult to identify. Blood traits measured after 14 d of feeding the experimental diets indicated an acidifying shift of the acid-base balance when the level of free AA inclusion was increased from 71 to 87 g/kg of diet, which was one AA substitution increment higher than the AA substitution that caused reduced ADG and ADFI. This may have been related to an affected acid-base balance on performance during the first days after diet change. Such an impact would have been undetectable when an affected acid-base balance was compensated by d 14 after diet change while days with lower performance were within the period of performance determination.

However, the study by Ibrahim et al. (2024a) did not investigate effects of diet change on performance and the acid-base balance within the first days after diet change. Effects of diets almost identical to those investigated by Ibrahim et al. (2024a) were investigated on the first days after diet change in a subsequent experiment. Performance, N balance measurements, and plasma free AA concentrations of this subsequent experiment were published previously (Ibrahim et al., 2024b). Ibrahim et al. (2024b) showed that diet change instantly led to increased concentrations of most free AA in the blood plasma and immediate changes in N utilization. Adaptations in N utilization and plasma free AA concentrations mainly occurred within 7 d after the diet change.

The objective of this communication is to determine metabolic adaptations on the acid-base balance and to reveal impacts on the metabolism of broiler chickens after change to diets containing high free AA levels. Blood samples obtained in the course of an experiment published previously (Ibrahim et al., 2024b) were used. Blood traits related to the acid-base balance were analyzed and an untargeted metabolomics assay was conducted. We hypothesized that a shift in the acid-base balance occurs immediately after a change to diets with high free AA levels. We further hypothesized that an untargeted metabolomics assay reveals physiological pathways affected by free AA inclusion, which would offer new explanations for reduced performance at high free AA inclusion.

## MATERIALS AND METHODS

The experiment was approved by the Regierungspräsidium Tübingen, Germany (permit number: HOH63/21_460a) and carried out according to German animal welfare legislation.

### Experimental Diets

A starter diet and 3 experimental diets were mixed at the certified feed mill of the Agricultural Experimental Station of the University of Hohenheim. The starter diet contained 80 g SPI/kg with 164 g CP/kg. One of the experimental diets was almost identical to the starter diet (**0FAA**). In the other diets, prececally digestible AA from SPI was substituted at 50% (**50FAA**) or 100% (**100FAA**) with a mix of free AA containing all 20 proteinogenic AA see (Ibrahim et al. 2024b). Digestible AA in the SPI was determined in a previous experiment (Ibrahim et al., 2024a). The digestible amounts of Asn+Asp and Gln+Glu provided by SPI were substituted with 50/50 mixes of Asp/Asn and Glu/Gln, respectively. The basal mix of all diets was mainly based on corn and casein. Free AA (19.8 g/kg) were added to the basal mix to achieve the intended AA concentrations. Concentrations of essential AA in all diets were calculated at 105% of the recommendations by the Gesellschaft für Ernährungsphysiologie (1999), and the concentrations of nonessential AA were chosen based on previous studies (Siegert et al., 2015; Hofmann et al., 2019, 2020).

The starter diet was pelleted using a 2-mm die while the other diets were pelleted through a 3-mm die without steam. Trying to overcome technical issues of producing the 3-mm pellets, soybean oil was added to the readily prepared mixtures, resulting in lower proportions of other ingredients so that AA concentrations were at a level of 103% of the recommendations. Pelleting went smoothly after configuration settings of the pelleting machines were fixed. The calculated AA values in the diets were confirmed by the results of the AA analysis of the diets (Ibrahim et al. 2024b). The dietary electrolyte balance was 145, 109, and 65 mEq/kg DM for 0FAA, 50FAA, and 100FAA, respectively. Concentrations of all nutrients reported herein are on an 88% DM basis unless otherwise stated.

### Birds and Experimental Procedures

One-day-old male Ross 308 broiler hatchlings were obtained from a commercial hatchery (Brüterei Süd ZN der BWE-Brüterei Weser-Ems GmbH & Co. KG, Regenstauf, Germany). Birds were held in 2 floor pens (2 m × 6 m) on dedusted wood shavings up to d 6 post-hatch. Three hundred and fifteen birds were distributed to 21 metabolism units (2 m × 1 m × 1 m) on a mesh-wired floor on d 6 to achieve an equal mean bird weight in each unit. Each diet was tested in 7 metabolism units and 15 birds each. Feed and water were offered for ad libitum consumption throughout the experiment. All birds received the starter diet until d 7 post-hatch when diet change occurred. The 3 experimental diets were then fed from d 7 to 21 post-hatch. Lighting was continuous during the first 3 d after placement to the metabolism units, followed by an 18 h light and 6 h dark cycle until the end of the experiment. The temperature was set at 34°C for the first 3 d post-hatch and was gradually decreased to about 21°C at the end of the experiment.

Birds were allocated to the metabolism units on d 6 post-hatch, 1 d before changing to the experimental diets to avoid reallocation stress at the beginning of the experimental phase. Additionally, birds were individually tagged on d 6 post-hatch to allow for a predefined euthanasia selection scheme to avoid impacts of a selection bias. Trunk blood was sampled by throat cut after anesthesia by a blunt blow on the head according to the directive 2010/63/EU. About 8 ml of blood was collected in lithium heparin tubes. An amount of 0.1 mL of blood was used to analyze the traits related to the acid-base balance and the remainder of the blood was centrifuged at 4°C and 1,500 *g* for 10 min and then the plasma was frozen at −80°C. Two birds were sampled from 7 units on d 7 post-hatch prior to change to the experimental diets, resulting in 14 baseline samples. On d 1, 2, 4, 7, and 14 after diet change, 2 birds per each unit were sampled, resulting in 14 samples per diet and day. Blood traits related to the acid-base balance were analyzed on all sampling d, that is, 0, 1, 2, 4, 7, and 14 after diet change, whereas the metabolomics analysis was performed using the samples of d 1, 7, and 14 after diet change.

### Chemical Analyses

Nutrient concentrations of the diets were analyzed as described in the accompanying communication (Ibrahim et al., 2024b). Traits related to the acid-base balance were determined using the i-STAT Alinity system (Abaxis Inc., Union City, CA) fitted with EC8+ cartridges. This included pH, carbon dioxide partial pressure (**PCO_2_**), total carbon dioxide (**TCO_2_**), bicarbonate (**HCO_3_**), base excess, sodium (**Na**), potassium (**K**), chloride (**Cl**), anion gap, and glucose.

### Untargeted Metabolomics Analysis of Blood Plasma

The metabolomics analysis was performed using Liquid Chromatography-Electrospray Ionization-Tandem-Mass Spectrometry (**LC-ESI-MS/MS**). Sample preparation included precipitation of intact proteins from blood plasma. 680 µl methanol was added to 170 µL of blood plasma and vortexed for 30 s. Proteins were precipitated by incubation of the samples for 2 h at −20°C. Samples were then centrifuged at 16,000 *g* for 5 min. The supernatants were transferred to 1.5 mL Eppendorf tubes and evaporated under an N stream in an XcelVap concentrator (Biotage, Uppsala, Sweden). Dried samples were dissolved in 100 µL water/methanol (1:1, v/v) for LC-MS/MS analysis.

The LC-ESI-MS/MS analysis was performed on a 1,290 UHPLC system (Agilent, Waldbronn, Germany) coupled to a Q-Exactive Plus Orbitrap mass spectrometer equipped with a heated electrospray ionization source (Thermo Fisher Scientific, Bremen, Germany). Analyte separation was achieved by a reversed-phase HSST3 C18 column (2.1 × 150 mm, 1.8 μm particle size, Waters GmbH, Eschborn, Germany). The column temperature was maintained at 40°C. 5 µL of each sample was injected. Mobile phase A was 0.2% formic acid and mobile phase B was acetonitrile with 0.2% formic acid. A constant flow rate of 0.3 mL/min was used and the gradient conditions were as follows: 0 to 3 min 1% B, 3 to 14 min 1% B-99% B, 14 to 18 min 99% B, 18 to 19 min 99% B-1% B, and re-equilibration at 0% B for 5 min. The total run time was 24 min.

The heated electrospray ionization source was operated in positive and negative ion mode, with a spray voltage of 5 kV in positive and negative ion mode. The ion transfer capillary temperature was set to 350°C. The sweep gas and auxiliary pressure rates were set to 60 and 20, respectively. The S-Lens RF level was 50%, and the auxiliary gas heater temperature was 150°C. Mass spectra were acquired within the mass range of 70 to 1,050 m/z at a resolution of 70,000 FWHM using an Automatic Gain Control target of 1.0 × 10^6^ of and 100 ms maximum ion injection time. For the acquisition of quantification data, the instrument was operated in the MS-only mode. Data dependent MS/MS spectra for the identification of metabolites were acquired in quality control samples only. MS/MS spectra were generated within a mass range of 200 to 2,000 m/z for the 5 most abundant precursor ions with a resolution of 17,500 FWHM using an Automatic Gain Control target of 2.0 × 10^5^, 100 ms maximum ion injection time and a stepped collision energy of 15, 30, and 45. Sample analysis was started with the injection of a solvent blank solution used for background subtraction. Samples were injected in a randomized order. Quality-control samples (a mixture of all samples in equal proportions) were injected before the first sample, after every 15th sample analysis, and after the last sample. Xcalibur version 4.4.16.14 and Compound Discoverer 3.3. software (Thermo Fisher Scientific, San Jose, CA) were used for data analysis, sum formula calculation and compound identification of metabolites. Molecular masses (m/z values), calculated sum formulas and fragmentation spectra of individual features were matched to references from compound databases (HMDB, ChemSpider, Lipid Maps, and PubChem) and spectral libraries (m/z cloud and HMDB). The assignment of metabolites was further verified by theoretical fragmentation using the Fragment Ion Search (FISh) scoring node in Compound Discoverer.

### Statistical Analysis

Blood traits were statistically analyzed by one‐way ANOVA using the MIXED procedure of SAS (Version 9.4, SAS Institute, Cary, NC) after confirming that BW as a covariate in a statistical analysis had a negligible impact. The individual birds were considered as the experimental unit after verifying that groups had no effect when groups were included in the statistical model as a random effect. The statistical model was:

(1) $$y_{ij}=\alpha +\text{tr}t_{i}+\text{bloc}k_{j}+e_{ij}$$

Where *y_ij_* is the dependent trait, *α* is the overall mean, trt*_i_* is the fixed effect of treatment *i* (0FAA, 50FAA, 100FA), block*_j_* is the random effect of block *j* (1–7), and e*_ij_* is the residual error. The random block *j* effect was included if model accuracy was improved, as indicated by the Akaike Information Criterion. Statistical significance was set at *P* < 0.050.

All downstream multivariate and univariate statistical analyses of normalized peak areas of the metabolomics approach were performed with MetaboAnalyst 5.0 software (Chong et al., 2019). The processed data were subjected to principal component analysis (**PCA**). One-way ANOVA according to the model specified in Eq. 1 was performed separately for each day to investigate the treatment effects. Data were subjected to partial least square analysis (**PLS-DA**) and the single variable importance in projection (**VIP**) for each day.

## RESULTS

### Acid-base Balance

**Figure 1:**
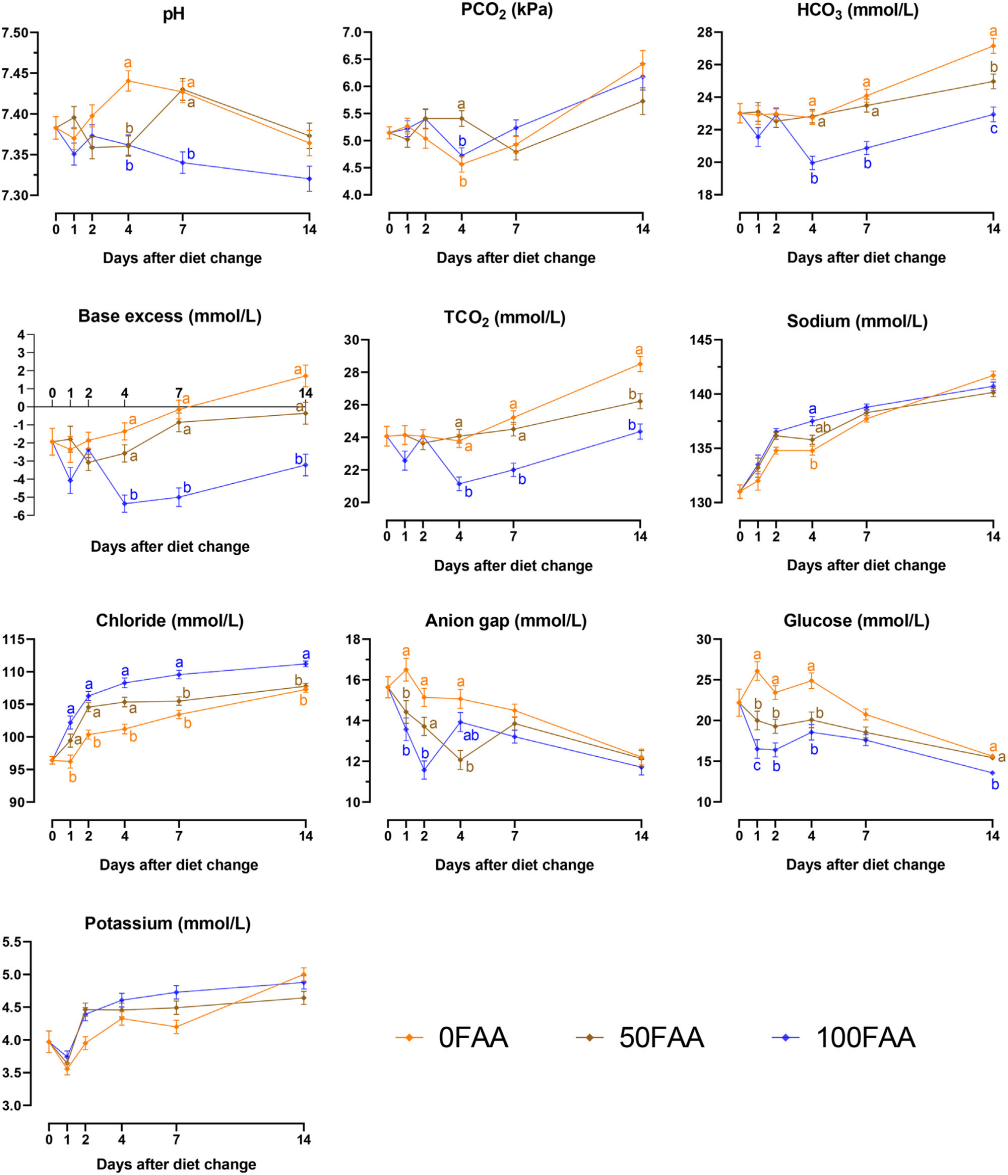
Effects of incremental substitution of digestible amino acids from 80 g soy protein isolate/kg in one diet (0FAA) with 50% (50FAA) or 100% (100FAA) free amino acids on selected blood traits related to the acid-base balance (PCO_2_, carbon dioxide partial pressure; HCO_3_, bicarbonate; base excess; TCO_2_, total carbon dioxide; potassium; sodium; chloride; anion gap; glucose) of broiler chickens (n = 14 birds/treatment, samples on d 0 were taken before diet change). Treatments within a day without a common superscript differ significantly (*P* < 0.050). Error bars indicate the pooled standard error of each day.

Blood pH decreased for 50FAA and 100FAA diets on d 4 after diet change (Figure 1, *P* ≤ 0.019) and for the 100FAA diet on d 7 after diet change (*P* ≤ 0.005) compared to 0FAA. Feeding the 50FAA diet increased blood PCO_2_ on d 4 after diet change (*P* = 0.047) compared to the other diets. On d 4, 7, and 14 after diet change, blood HCO_3_, base excess, and TCO_2_ were lower for 100FAA (*P* ≤ 0.006) compared to 0FAA and 50FAA. Compared to 0FAA, blood Cl was increased on d 1, 2, 4, 7, and 14 after diet change for 100FAA (*P* < 0.001) and on d 1, 2, and 4 after diet change for 50FAA (*P* ≤ 0.030). Blood Na was increased for 100FAA on d 4 after diet change (*P* = 0.007) compared to the other diets with lower free AA levels. High free AA levels compared to 0FAA decreased the anion gap for 50FAA and 100FAA on d 1 after diet change, for 100FAA on d 2 after diet change, and for 50FAA on d 4 after diet change, respectively (*P* ≤ 0.012). Similarly, 50FAA and 100FAA diets decreased blood glucose on d 1, 2, and 4 after diet change (*P* ≤ 0.035), and on d 14 after diet change for 100FAA diet (*P* ≤ 0.007) compared to 0FAA. Plasma K concentrations were not affected by treatment.

### Untargeted Metabolomics

**Figure 2:**
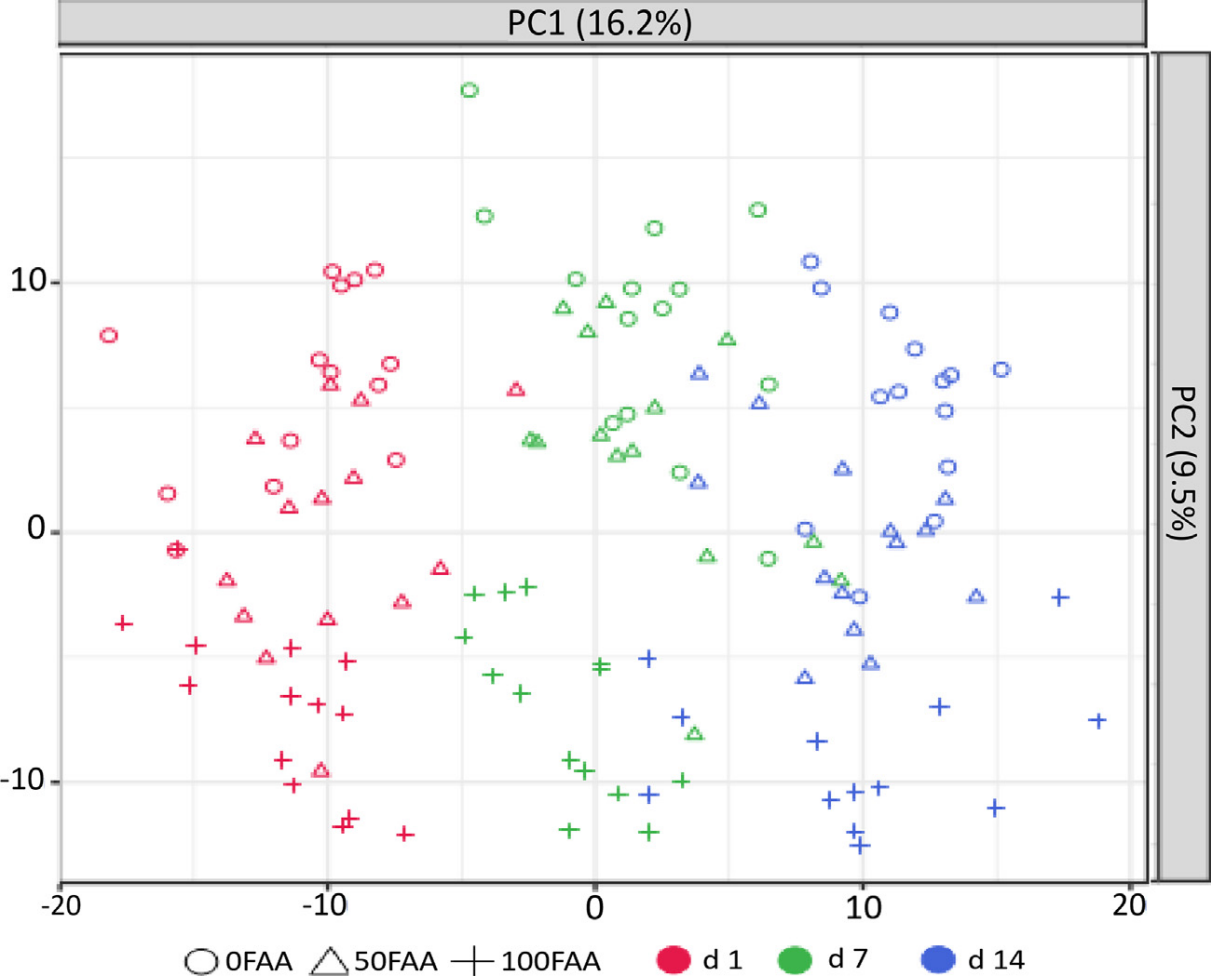
Effects of incremental substitution of digestible amino acids from 80 g soy protein isolate/kg in one diet (**0FAA**) with 50% (**50FAA**) or 100% (**100FAA**) free AA on the 2-way principal component analysis scores plot of the untargeted metabolomics approach in the blood plasma of broiler chickens sampled on days (**d**) 1, 7 and 14 after diet change.

**Figure 3:**
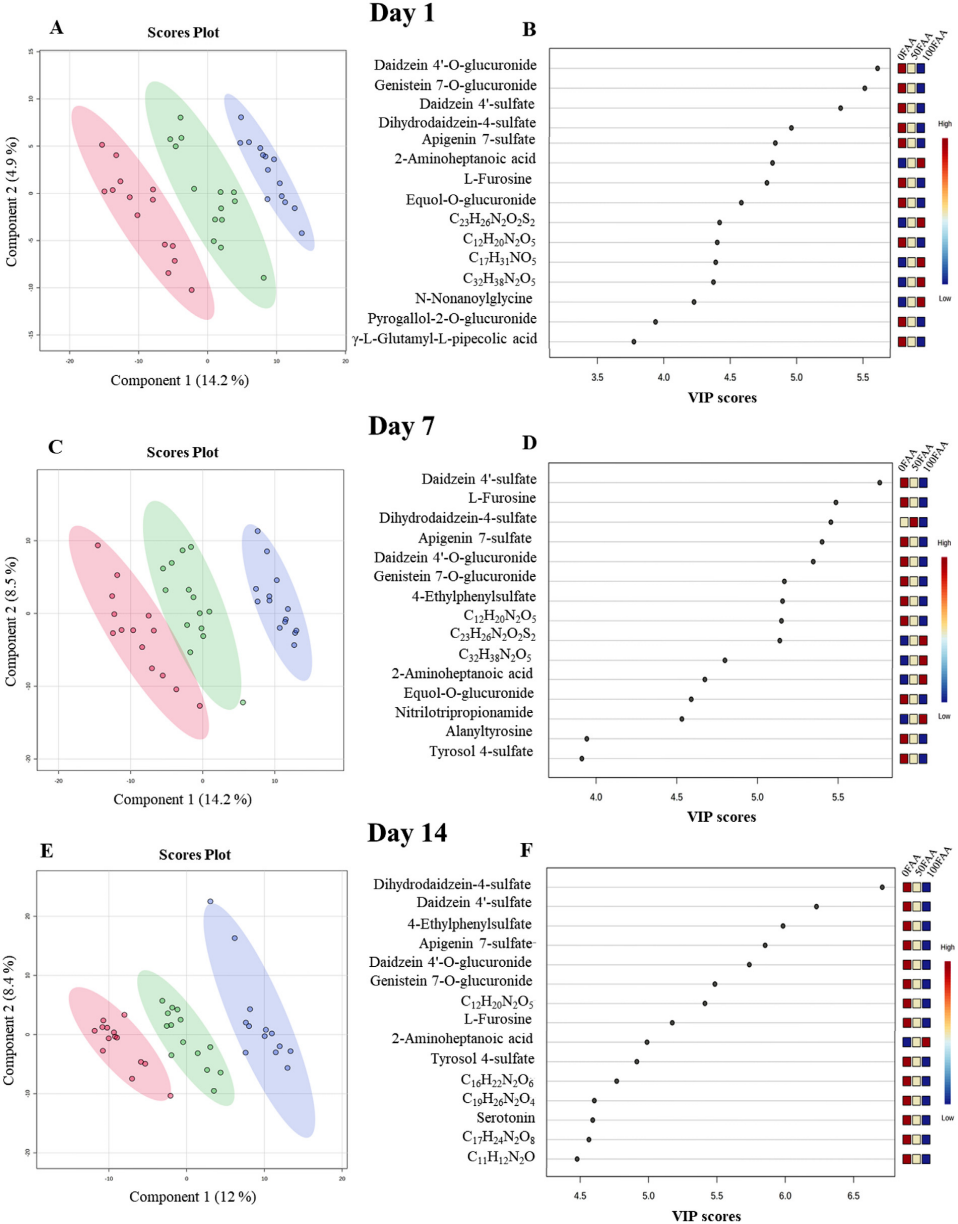
Partial least squares discriminant analysis (**PLS-DA**) scores plots of the substitution of digestible amino acids from 80 g soy protein isolate/kg in one diet (**0FAA**, in red) with 50% (**50FAA**, in green) or 100% (**100FAA**, in blue) of free amino acids on d 1 (panel A), 7 (panel C), and 14 (panel E) after diet change. Variable importance in projection (**VIP**) plots of the metabolites most affected by the diets are presented separately for d 1 (panel B), 7 (panel D), and 14 (panel F) after diet change. Metabolites are presented as sum formulas if identification of metabolites was not definite. The colored scale from blue to red indicated the relative abundance of metabolites from low to high.

The untargeted metabolomics assay detected a total of 4,832 features after subtraction of background features and features eliminated by the normalization process. The library matching confirmed the identity of 2,876 metabolites with high confidence. PCA analysis of the confirmed metabolites revealed clustering according to both sampling days and treatments, with 50FAA being intermediate to 0FAA and 100FAA (Figure 2). Using one-way ANOVA for each sampling d, 602, 463, and 302 metabolites were determined to be significantly affected by treatment on d 1, 7, and 14 after diet change, respectively (*P* < 0.050; Supplementary Table 1). The clustering of confirmed metabolites by diets with 50FAA being intermediate to 0FAA and 100FAA was also observed when PLS-DA scores plots were created separately for each sampling day (Figure 3). The first 2 components of PLS-DA together covered 19.1, 22.7, and 20.4% of the variation for d 1, 7, and 14 after diet change, respectively. The VIP scores indicated the 15 most important metabolites contributing to the separation between the treatments (Figure 3). Eight metabolites were consistently observed in the VIP scores on d 1, 7, and 14 after diet change. Five of those metabolites were classified as the flavonoids genistein 7-O-glucuronide, daidzein 4′-sulfate, daidzein 4-O-glucuronide, dihydrodaidzein-4-sulfate, and apigenin 7-sulfate. The remaining metabolites, including C_12_H_18_N_2_O_4_ (most likely l-furosine), C_7_H_15_NO_2_ (most likely 2-aminoheptanoic acid), and C_12_H_20_N_2_O_5_ were associated with a diverse range of biochemical classes or could not be classified using the databases.

## DISCUSSION

The results indicated that some traits related to the acid-base balance were affected by AA substitution immediately after diet change. For 100FAA, metabolic acidosis was determined from d 4 to 7 after diet change. This was indicated by decreased pH, HCO_3,_ and base excess, as well as increased Cl in the blood (Patience, 1990; Montesinos and Ardiaca, 2013; Macalintal et al., 2020) on d 4 and 7 after diet change. Respiratory acidosis was evident for 50FAA on d 4 after diet change, as indicated by decreased pH and increased PCO_2_ without affecting HCO_3_ in the blood (Olanrewaju et al., 2013). As the pH values were significantly affected on d 4 and d 4 to 7 after diet change for 50FAA and 100FAA, respectively, the acute response phase on the acid-base balance was on these days after diet change. Thus, the first hypothesis that an acidifying shift in the acid-base occurs immediately after diet change was accepted, but the relevant phase started on d 4 and this persisted until d 7 after diet change.

Potential reasons for the acidifying shift in the acid-base balance include AA oxidation or the type of free AA. As described in the accompanying communication (Ibrahim et al., 2024b), the N utilization efficiency was immediately reduced for 50FAA and 100FAA compared to 0FAA and it took 4 d after diet change until these differences in N utilization efficiency disappeared. A reduced N utilization efficiency for diets with similar prececally digestible AA concentrations likely results from increased AA degradation. The consequences of AA degradation on the acid-base balance depend on the AA being oxidized (Patience, 1990), which is difficult to predict: The oxidation of dicarboxylic anionic AA, such as Asp and Glu, tends to cause alkalosis. In contrast, oxidation of sulfur AA, such as Met and Cys, is acidic and would contribute to acidosis. However, May et al. (1986) found that AA oxidation resulted from metabolic acidosis in rats. Possibly, AA degradation intensified an acidifying shift in the acid-base balance in the present study. Nonetheless, N utilization efficiency was not affected by treatment from d 4 after diet change onwards in the present experiment (Ibrahim et al. 2024b) and the acute response phase of the acidosis became manifest on the fourth day after diet change. This indicated that the relevance of AA oxidation for the acid-base balance was low in the present study or that such effects were compensated by other influences. A shift of the acid-base balance towards acidity might also occur when peptide-bound AA were substituted with free AA by HCl supply from l-Lys·HCl. The supply of free l-Lys·HCl to the 100FAA diet added approximately 1.6 g HCl/kg DM. Whether the HCl provided by l-Lys·HCl was relevant for the acid-base balance warrants further investigation.

Following the acute response phase, the adaptation to the acidosis took longer for 100FAA than the adaptation to 50FAA. For 50FAA, blood pH was not different from 0FAA on d 7 after diet change, while it took until d 14 after diet change for 100FAA until blood pH was not significantly different to 0FAA. After the acute response phase, decreased HCO_3_ and base excess, as well as increased Cl in the blood, indicated that the birds still dealt with an acid load (Atherton, 2009; Montesinos and Ardiaca, 2013), but compensatory mechanisms enabled them to maintain blood pH. An adaption of ammonia excretion probably contributed to maintaining blood pH. Ammonia formation in the kidneys and ammonia excretion via the urine are known to represent an adaptive response to excrete acids and to maintain the acid-base balance (Craan et al., 1982; Hamm and Simon, 1987; Patience, 1990; Weiner and Hamm, 2007). Ammonia excretion increased with rising levels of free AA throughout the experimental period and was higher for 100FAA compared to 50FAA, as described in the accompanying communication (Ibrahim et al., 2024b). The results of the current study are consistent with the results of a previous experiment using almost identical diets (Ibrahim et al., 2024a), where a compensated acidosis was indicated 14 d after diet change. A further indication of adaptation to high levels of dietary free AA was provided by blood plasma metabolites. The decreasing number of metabolites determined by one-way ANOVA affected by treatment on d 1, 7, and 14 after diet change (602, 463, and 302 metabolites, respectively) may point to an adaptation to high dietary free AA levels.

The acidifying shift in the acid-base balance may have caused the reduced ADFI, particularly in the first days after diet change. As described in the accompanying communication (Ibrahim et al., 2024b), ADFI decreased with increasing AA substitution on the first day after diet change and remained on the same level on the second day after diet change. From d 4 after diet change onwards, ADFI was on the level of the performance objective of the breeding company (Aviagen, 2019) for 0FAA and 50FAA, but 100FAA continued to cause lower ADFI. It is possible that high ammonia formation to counter a metabolic acidosis caused elevated blood ammonia concentrations in addition to the observed higher ammonia excretion. A correlation between blood ammonia concentrations and ADFI was found in broiler chickens (Namroud et al., 2008) and high blood ammonia concentrations in humans as well as rats were associated with anorexia (Noda and Chikamori, 1976; Walker, 2009). The birds in the present study may have tried to attenuate the impacts of an acid load by avoiding the diet as the cause for the acid load. Particularly the low ADFI on the first 2 d after diet change may have contributed to the observed time lag between diet change and the acute response phase of the acidosis. The interpretation of lower ADFI as a reaction to avoid the acid load by the diet can also explain the lower ADFI of 100FAA compared to 0FAA on d 14 after diet change, although the acute response phase of the acidosis was over. The lower capacity for feed intake due to less body weight gain may also have contributed to the lower ADFI of 100FAA than 0FAA on d 14 after diet change.

There are indications that the 50FAA diet with ∼54 g free AA/kg was close to the maximum free AA inclusion in the diets that can be achieved without affecting performance under the conditions of our study. The ADG of 50FAA diet was not significantly different to 0FAA throughout the experimental period on each observation day (Ibrahim et al., 2024b). This was consistent with the results by Ibrahim et al. (2024a) that the maximum free AA inclusion in diets was found between 54 and 71 g free AA/kg. However, respiratory acidosis, as indicated on d 4 after diet change, usually shows up rapidly in case of an affected acid-base balance, and metabolic acidosis is determined when the impact on the acid-base balance is more severe (Atherton, 2009). Hence, the additional acid load to that of 50FAA that would have caused the metabolic acidosis was probably low.

The untargeted metabolomics approach did not indicate physiological pathways affected by free AA inclusion. The number of significantly affected and classified metabolites was small overall and the affected metabolites were structurally too different to conclude on a significant influence on a defined physiological pathway. A consistency that could be observed from the metabolomics data was a decreasing concentration of isoflavones and other flavonoids in the blood plasma, including glucuronide and sulfate metabolites of genistein and daidzein, with increasing substitution of SPI with free AA. Such isoflavones are typically found in soy products (Franke et al., 1994) and the corresponding glucuronides and sulfates are typical metabolic products produced in the liver (Muñoz-González et al., 2019). Hence, the decrease in concentrations of flavonoid metabolites in the blood plasma upon increasing substitution of soy-based feed ingredients with free AA indicated the validity of the untargeted metabolomics approach. In turn, the outcomes suggest that AA substitution had only a minor influence on physiological pathways. Hence, the second hypothesis that an untargeted metabolomics assay reveals physiological pathways affected by free AA inclusion was rejected. Nonetheless, some very hydrophilic metabolites, like small organic acids, were not detected in our metabolomics approach, since they elute in the void volume of the reversed-phase chromatography. The reversed-phase chromatography approach mainly detects non-polar metabolites, which include metabolites of the protein metabolism, while highly polar metabolites cannot be detected (Patti, 2011). Therefore, we cannot exclude that treatments may have caused changes in concentrations of very hydrophilic metabolites.

In conclusion, the current study showed that the impact on the acid-base balance was most pronounced on d 4 to 7 after change to diets containing high free AA levels. In addition, compensated acidosis was determined on d 7 after diet change for 50FAA and d 14 after diet change for 100FAA, respectively. Thus, adaptations to 100FAA took longer than for 50FAA. The untargeted metabolomics assay was sensitive to show that substituting digestible peptide-bound AA from SPI with free AA led to decreasing concentrations of compounds in the blood plasma that are associated with plant-based feed ingredients; however, the approach did not reveal physiological pathways that were affected by AA substitution.

## DISCLOSURES

The authors declare no conflicts of interest.

## Appendix Supplementary materials

Image, application 1

## Footnotes

Supplementary material associated with this article can be found in the online version at doi:10.1016/j.psj.2024.103956.
